# A screen for inducers of bHLH activity identifies pitavastatin as a regulator of p21, Rb phosphorylation and E2F target gene expression in pancreatic cancer

**DOI:** 10.18632/oncotarget.18587

**Published:** 2017-06-21

**Authors:** Nicholas Villarino, Lia Signaevskaia, Jaco van Niekerk, Rachel Medal, Heejung Kim, Reyhaneh Lahmy, Kathleen Scully, Anthony Pinkerton, Sangwun Kim, Andrew Lowy, Pamela Itkin-Ansari

**Affiliations:** ^1^ Development, Aging and Regeneration Program, Sanford Burnham Prebys Medical Discovery Institute, La Jolla, CA, USA; ^2^ Conrad Prebys Center for Chemical Genomics, Sanford Burnham Prebys Medical Discovery Institute, La Jolla, CA, USA; ^3^ Division of Gynecologic Oncology, Department of Obstetrics and Gynecology, Institute of Women's Life Medical Science, Yonsei University College of Medicine, Seoul, Republic of Korea; ^4^ Departments of Pathology and Surgery, Division of Surgical Oncology, Moores UCSD Cancer Center, University of California San Diego, La Jolla, CA, USA; ^5^ Department of Pediatrics, University of California San Diego, La Jolla, CA, USA

**Keywords:** bHLH proteins, pancreatic cancer, high throughput screening, drug discovery, statins

## Abstract

The average survival for patients with Pancreatic Ductal Adenocarcinoma (PDA) is merely 6 months, underscoring the need for new therapeutic approaches. During PDA progression, pancreatic acinar cells lose activity of the ClassI/II bHLH factors that regulate quiescence. We previously found that promoting transcriptional activity of the Class I bHLH factor E47 in highly aggressive PDA cells induced stable growth arrest *in vitro* and *in vivo*. To translate these findings for clinical utility, we developed a high throughput screening platform to identify small molecule inducers of Class I/II bHLH activity. A screen of 4,375 known drugs identified 70 bHLH activators. Prominent among the hits were members of the statin class of HMG-CoA reductase inhibitors, cholesterol lowering drugs that are also being evaluated in cancer. Studies with pitavastatin in primary patient derived tumor cells and established PDA lines, revealed dose dependent growth inhibition. At the molecular level, pitavastatin induced expression of the cyclin dependent kinase (CDK) inhibitor p21 in a cholesterol independent manner, blocked repressive phosphorylation of the Retinoblastoma tumor suppressor protein at CDK targeted sites, and reduced expression of E2F target genes required for progression through the G1/S boundary. Together, the data provide new insight into mechanisms by which statins constrain proliferation in cancer and establish the effectiveness of a novel screening platform to identify small molecules of clinical relevance in pancreatic cancer.

## INTRODUCTION

Pancreatic Ductal Adenocarcinoma (PDA) is an almost uniformly lethal cancer and is projected to be the second leading cause of cancer deaths in the United States by 2030 [1]. The stark statistics highlight the need for a deeper understanding of PDA pathophysiology and for novel therapeutic approaches.

PDA is fueled by mutations in four primary drivers; activating mutations of *KRAS* are accompanied by mutations or silencing of SMAD4, TP53, and CDKN2A that encodes the cyclin dependent kinase (CDK) inhibitor p16INK4A [2]. The loss of wild-type p53 leads to chromosomal instability and alterations in transcription of its target genes, including reduced expression of the CDK inhibitor CDKN1A/p21 [3]. In contrast to p53, the Retinoblastoma (Rb) tumor suppressor is rarely mutated in PDA, but as a consequence of p16 and p21 loss, CDKs phosphorylate Rb at specific serine residues, rendering it inactive [4]. Ultimately, the repression of Rb activity enables E2F transcription of genes that promote proliferation.

During PDA progression, tumor-initiating cells repress the expression of the pancreas-restricted transcription factors, PTF1a and MIST1 that normally constrain growth and promote the acinar differentiation program [5–7]. PTF1a and MIST1 belong to the tissue restricted subclass (Class II) of basic helix-loop-helix (bHLH) transcription factors. In order to bind to cognate E-box DNA sites in target genes, Class II bHLH proteins assemble as homodimers or in heterodimer complexes with more commonly expressed Class I bHLH proteins, e.g. E47 [8]. The bHLH family is also subject to negative regulation by the inhibitor of DNA binding (ID) family of proteins (ID 1-4 in humans). ID proteins lack a DNA binding domain and form nonfunctional dimers with bHLH proteins [9, 10]. We and others have shown that ID protein expression is elevated in human PDA [11–16]. Moreover, we established that ID3 alone was sufficient to induce proliferation in normally quiescent human pancreatic exocrine cells [11]. Conversely, we recently showed that restoring bHLH/ID balance in PDA by overexpression of E47 provoked rapid, p21 dependent, G1 arrest *in vitro* and *in vivo*. Intriguingly, growth arrest was accompanied by a striking degree of acinar reprogramming [17]. Thus, restored E47 bHLH activity in PDA functions as a genetic buffer that drives attenuation of powerful oncogenic cues. Having identified a genetic method for inhibiting PDA pathogenesis we sought to determine the potential for clinical translation of these findings.

Here we describe the development of a high throughput screening (HTS) platform to identify compounds that selectively induce Class I/II bHLH activity, as not all bHLH factors serve as tumor suppressors (e.g. c-myc). PDA cells were transduced with a luciferase reporter driven by multimerized E-boxes that are the preferred sequences for the Class I/II bHLH proteins. A screen of 4375 pharmaceuticals identified statins, a class of cholesterol lowering drugs, as potent activators of bHLH activity. We show that statins suppress growth across genomically diverse PDA lines. At the molecular level, statins promote p21 expression, Rb de-phosphorylation and repression of E2F target genes that control the G1/S checkpoint. Further, statins induced the bHLH proteins Dec-1 and MIST1 and upregulated the acinar enzyme trypsinogen in some lines.

Together, the data elucidate mechanisms by which statins blunt proliferation in PDA and establish an HTS assay for bHLH activity as a valuable tool for drug discovery in PDA.

## RESULTS

### Development of a high throughput screening platform for bHLH activity

We previously showed that increased expression of the Class I bHLH protein E47 was sufficient to induce quiescence and acinar differentiation in PDA cells [11, 17]. In order to extend these studies for translational potential we developed a screening platform to identify pharmacological inducers of Class I/II bHLH activity.

**Figure 1:**
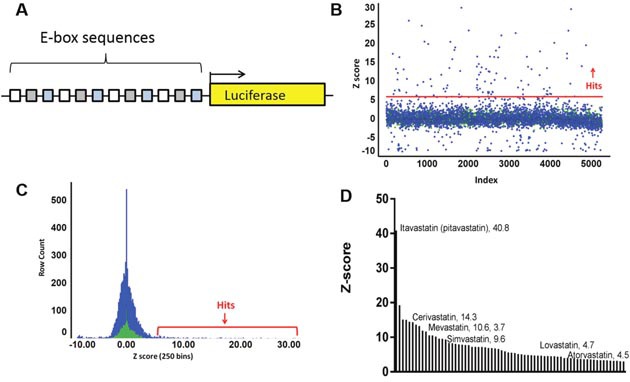
BHLH platform design and drug screen **(A)** The reporter vector consists of 12 bHLH binding elements (E-boxes), as 4 copies of 3 unique sequences, driving luciferase. **(B)** A screen of repurposed libraries yielded 271 hits above 3 standard deviations from the mean and 30 above 6 standard deviations from the mean (red line). **(C)** A histogram illustrates that the normal distribution peak from the compounds (blue) coincides with the DMSO peak (green). **(D)** Seventy unique hits were confirmed in triplicate and plotted in descending Z’, revealing enrichment for the statin class of compounds.

The canonical bHLH binding sequence, the ‘E-box’, is CANNTG. To enhance identification of activity from Class I and Class II bHLH factors, we employed a reporter consisting of the Class I/Class II preferred E-box sequences CACCTG, CAGCTG, and CATGTG. Each E-box was repeated 4 times in an alternating pattern, hereafter termed “E-box_luc” (Figure 1A and Supplementary Figure 1) [20]. Importantly, these E-boxes are not the preferred sequences for the bHLH/ZIP pro-growth factor c-myc that binds preferentially to CACGTG, nor for the bHLH/ZIP SREBP, that prefers the sequence ATCACCCCAC.

The bHLH reporter exhibited low baseline activity in all PDA cell lines tested, as predicted by diminished bHLH activity in pancreatic cancer, reported by us and others [5–7, 11]. To establish a positive control for the assay, we generated a derivative of the PANC1 line which stably expressed a tamoxifen inducible form of E47 in which E47 is fused to a modified estrogen receptor (PANC1/E47^MER^) [17–19]. To optimize induction of the bHLH reporter, a tamoxifen dose curve was generated in PANC1/E47^MER^ cells plated in 96 well format. The assay revealed that 2 μM to 8 μM tamoxifen optimally induced E-box-luc activity at 48 hours and 4uM tamoxifen was chosen for further use. Due to reports that the pH indicator phenol red can interfere with luciferase readout, RPMI media with and without phenol red was also evaluated, with the finding that 5 mg/L phenol red did not perturb luciferase activity (Supplementary Figure 2A).

The assay was miniaturized to a 384 well format by optimizing plating densities (Supplementary Figure 2B). To generate a Z’ we compared bHLH reporter activity in parental PANC1 cells versus PANC1/E47^MER^ cells treated with 4 μM tamoxifen. Luciferase activity was 2526±533 for PANC1 cells and 617686±84492 in the positive controls wells (Supplementary Figure 3), yielding a Z’ of 0.585. Importantly, parental PANC1 cells, not PANC1/E47^MER^ cells, were employed for the subsequent screen, because we previously determined that the modified estrogen receptor tag is susceptible to activation by estrogenic and tamoxifen-like molecules, leading to biologically irrelevant hits [19, 21].

### Screen of 4375 pharmaceuticals identifies the statin class of drugs as inducers of bHLH activity

Five libraries of known pharmaceuticals, consisting of a total of 4375 compounds, were selected for screening in an effort to maximize rapid clinical translation of the findings. Compounds were tested at 10μM for 48 hours, and each assay plate contained 64 vehicle control wells. Hits from the screen were evenly distributed throughout the plates (Figure 1B, Supplementary Figure 4) and a histogram shows that the majority of compounds formed a normal distribution overlapping with DMSO controls (Figure 1C). The screen identified 271 compounds with values greater than 3 standard deviations from the mean of 64 DMSO treated wells per plate. Moreover, twenty-one of the hits were duplicated in 2 libraries and two of the compounds were replicated in 3 libraries, thus providing early validation of the screen and yielding 246 unique hits.

In a confirmatory screen each of the 246 hits were assayed in triplicate, resulting in 70 unique hit compounds (Figure 1D). Among these, thirty compounds exhibited a Z’ score above 6, and 12 compounds had a Z’ above 10. The most significant hit was pitavastatin (itavastatin), which registered a Z’=40.8 (Figure 1D). Pitavastatin belongs to the statin class of cholesterol lowering drugs which target hydroxymethylglutaryl coenzyme A reductase (HMG-CoA Reductase or HMGCR), the rate-limiting enzyme in de novo cholesterol synthesis [22]. Five additional statins were also among the confirmed hits, suggesting a conserved mechanism of action within the chemotype.

### Statins upregulate p21 and repress genes associated with proliferation in patient derived PDA tumor cells and established lines

**Figure 2:**
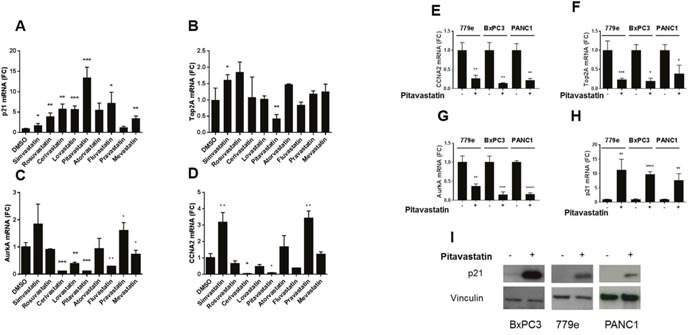
Statins regulate expression of cell cycle genes **(A-D)** PANC1 cells treated with 10 μM of each of 9 statins for 48 hours were harvested for RT-qPCR for CCNA2, TOP2A, AURKA, and p21, shown as fold change over DMSO treated cells. **(E-I)** To extend the studies to additional cell lines, PANC1, BxPC3 and a low passage line derived from a patient's tumor, 779e, were treated with 10uM pitavastatin for 48 hours. RT-qPCR analysis of **(E)** CCNA2, **(F)** TOP2A and **(G)** AURKA revealed significantly reduced mRNA expression in all three lines. Conversely the tumor suppressor p21 was significantly induced by pitavastatin at the **(H)** mRNA level, and **(I)** the protein level in all three lines. Data are represented as mean +/−SD, *p≤0.05, **p≤0.01, ***p≤0.001, ****p≤0.0001.

Previously we demonstrated that restoring bHLH activity in PDA cells by E47 overexpression significantly downregulated the cell cycle activators Cyclin A (CCNA2), Topoisomerase 2A (Top2A), and Aurora kinase A (AurkA). To determine whether statins regulate the cell cycle through these effectors, we investigated each of the nine statins. PANC1 cells were treated with 10μM of each statin. RT-qPCR analysis revealed that pitavastatin significantly reduced mRNA levels of CCNA2, Top2A and AurkA, and that cerivastatin reduced expression of AurkA and CCNA2 (Figure 2). Multiple statins also regulated cell cycle genes at 5uM (Supplementary Figure 5). In previous studies, we showed that E47 induced growth arrest required induction of the CDK inhibitor p21 [17]. Strikingly, 8 of 9 statins significantly induced p21 mRNA expression. Pitavastatin exhibited the most robust p21 induction, (p=0.00091) (Figure 2), and in a dose responsive manner (Supplementary Figure 5).

In tertiary screens we expanded the studies to include additional PDA lines. In contrast to PANC1, which harbors both a Kras mutation and wildtype SMAD4, the BxPC3 line has wildtype Kras but possesses a SMAD4 deletion. To maximize clinical relevance of the findings, we also employed 779e, a low passage primary PDA cell line recently derived from a patient tumor. At baseline the 779e line exhibits the highest expression of the epithelial marker E-cadherin and lowest levels of the mesenchymal marker vimentin (Supplementary Figure 6). Moreover, at baseline 779e cells also exhibit a 2 log increase in trypsin 2 mRNA relative to PANC1, and 19.5 fold more than BxPC3. Similarly, 779e cells express the highest level of the acinar maturation bHLH protein MIST1. Overall, 779e appears to be the most epithelial and acinar differentiated line, and PANC1 the least differentiated.

As observed in PANC1, pitavastatin treatment of BxPC3 and 779e cells also significantly reduced expression of Top2A, AurkA, and CCNA2, while inducing significant levels of p21 (Figure 2E-2H). Western blots showed that p21 protein expression mirrored that of the mRNA in all lines (Figure 2I). Thus, the regulation of cell cycle genes by pitavastatin is consistent across diverse genomic landscapes.

### Pitavastatin attenuates PDA cell growth in a dose responsive manner

**Figure 3:**
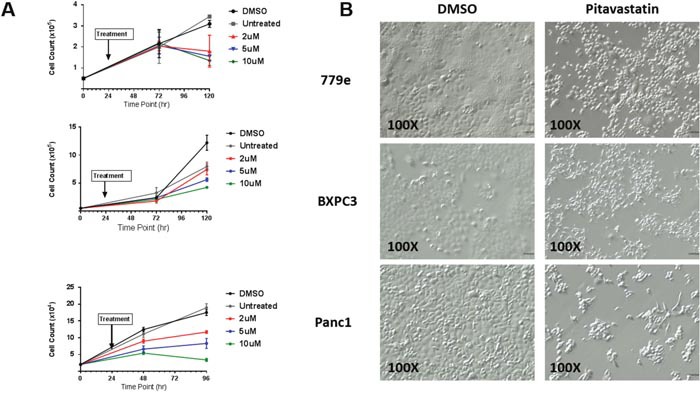
Pitavastatin attenuates PDA cell growth **(A)** BxPC3, PANC1 and 779e cells were treated with vehicle or pitavastatin (2 μM, 5 μM and 10 μM) 24 hours after plating. Replicate plates were counted at 72 and 96 or 120 hours after plating, revealing dose responsive growth inhibition. **(B)** Pitavastatin induced cytoskeletal alterations are evident in cells treated with 10 μM pitavastatin for 48hr. Bright field images are 100X original. Data are represented as mean +/−SD.

To examine the effect of pitavastatin on growth rate, PANC1, BxPC3, and 779e cells were treated with vehicle or 2, 5, and 10 μM pitavastatin (Figure 3A). Pitavastatin exerted a dose- and temporal-dependent growth inhibitory effect in all cell lines, with 10 μM pitavastatin halting proliferation in PANC1 and 779e cells. 779e exhibited the highest degree of drug sensitivity, with complete growth arrest at 2uM pitavastatin. PANC1 and BxPC3 cells resumed growth following removal of pitavastatin while 779e cells remained arrested (Supplementary Figure 7).

The inhibition of HMG-CoA reductase by statins is known to repress downstream isoprenoid synthesis, which is critical for maintenance of cytoskeletal integrity. We therefore investigated whether cytoskeletal irregularities were present in PDA cells treated with 10 μM pitavastatin. Similar to morphologic changes observed in some cancer cells treated with simvastatin, pitavastatin treated 779e cells adopted a circular morphology [23–26]. In contrast, a significant population of BxPC3 and PANC1 cells exhibited a fibroblast like morphology in which cells became elongated in a bipolar or multipolar fashion (Figure 3B). Simvastatin exerted little effect on overall PDA cell morphology (Supplementary Figure 8).

### Pitavastatin induced p21 expression and growth inhibition are SREBP1 independent

**Figure 4:**
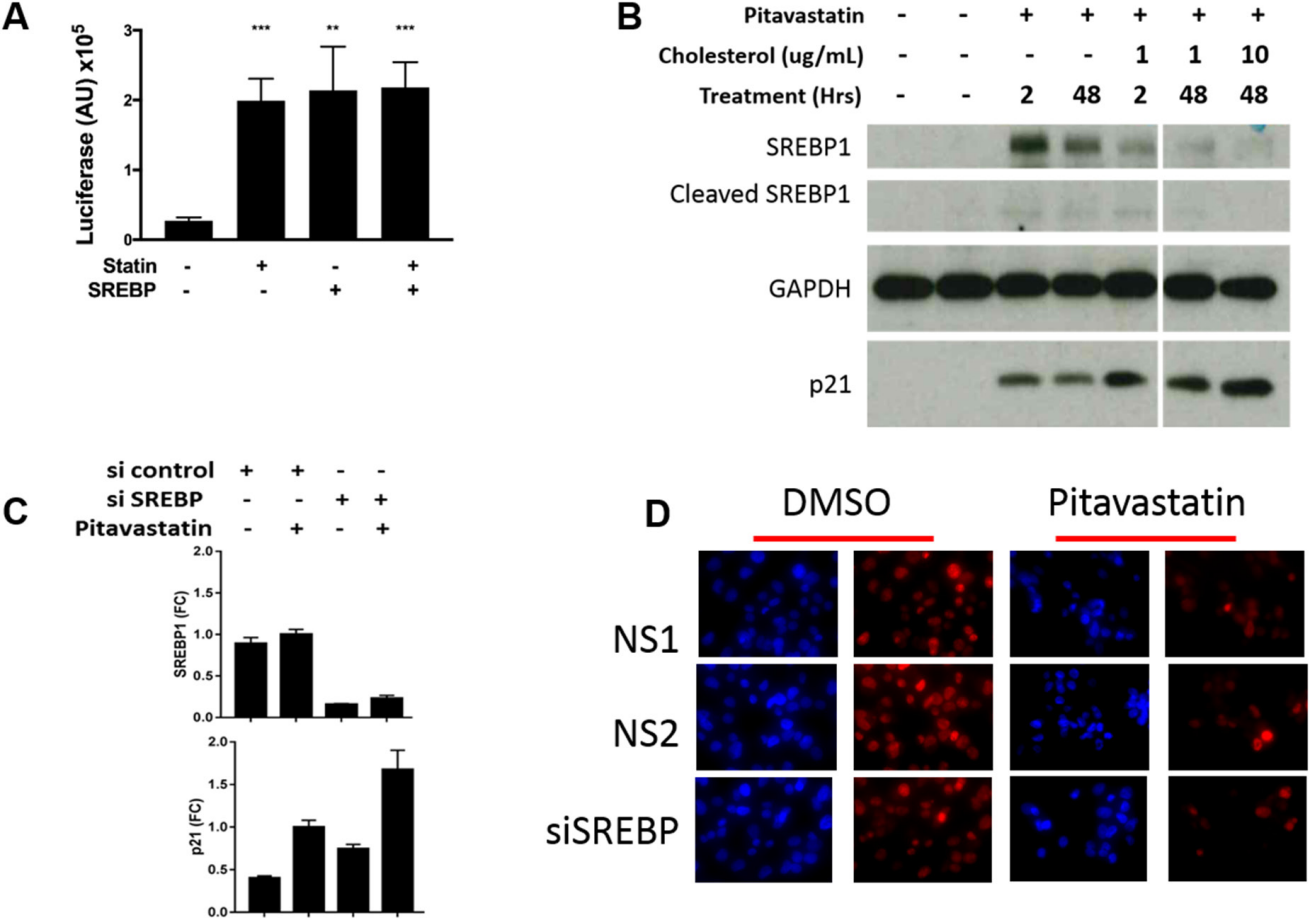
The role of SREBP1 in p21 expression and cell growth in PDA cells **(A)** PANC1 cells were transfected with an SREBP1 reporter containing multimerized SRE sites upstream of luciferase. Luciferase activity was measured 48 hours after treatment +/− pitavastatin and +/− a plasmid overexpressing SREBP1a. **(B)** Cells were treated with10μM pitavastatin for 48 hours and with 0, 1 or 10 μg/mL cholesterol for 2 or 48 hours. Lysates were collected for western blots for: SREBP1- full length and cleaved active form, p21 and GAPDH. **(C)** Cells were transfected with non-specific (NS) or SREBP1 specific siRNA in the presence or absence of pitavastatin (pita) and harvested for RNA extraction 72 hours later. **(D)** Concurrently cells were fixed and immunostained for Ki67 (red) and nuclei were stained with DAP1 (blue). Images are 200x. Data are represented as mean +/−SD.

When intracellular levels of cholesterol fall, the endoplasmic reticulum resident SREBP1 bHLH/ZIP transcription factors are cleaved and relocalized to the nucleus where they induce transcription of genes involved in raising intracellular levels of cholesterol. This in turn stimulates ingress of circulating cholesterol into cells, thus lowering serum levels of cholesterol. To evaluate SREBP1 activity in pitavastatin treated PDA cells, PANC1 cells were transduced with an SREBP1 luciferase reporter plasmid [27, 28]. Pitavastatin treatment induced SREBP1 reporter activity 7.3 fold above baseline (p<0.001), similar to SREBP1a overexpression in the absence of pitavastatin (7.9 fold, p<0.01) (Figure 4A). High reporter activity was accompanied by both elevated SREBP1 protein expression and cleavage to the active form (Figure 4B). To determine whether SREBP1 was required for p21 induction, PANC1 cells treated with 10 μM pitavastatin were co-treated with cholesterol to inhibit SREBP1 activation. Exposure to 10μg/mL cholesterol for 48 hours significantly repressed the pitavastatin-regulated SREBP1 expression and cleavage to the active form. Cholesterol treatment did not however inhibit pitavastatin induced p21 expression (Figure 4B). To further investigate its role, knockdown of SREBP1 was achieved using siRNA. Notably, 76.8% SREBP1 knockdown had no effect on pitavastatin induced p21 protein expression (Figure 4C) or Ki67 expression (Figure 4D). Together the data indicate that pitavastatin induced p21 expression and proliferative arrest in PDA occur through an SREBP1 independent mechanism.

### Pitavastatin induces expression of the hypoxia regulated bHLH protein Dec-1

**Figure 5:**
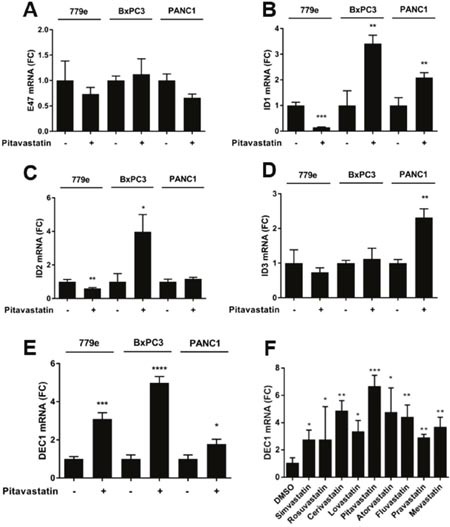
Pitavastatin induces DEC1 expression PANC1, 779e and BxPC3 cells were treated with pitavastatin or vehicle for 72hrs and analyzed by RT-qPCR for **(A)** E47, **(B)** ID1, **(C)** ID2, **(D)** ID3, and **(E)** DEC1. **(F)** DEC1 expression was also assayed in PANC1 cells treated with all 9 statins. Data are shown as fold change over DMSO treated cells. Data are represented as mean +/−SD, *p≤0.05, **p≤0.01, ***p≤0.001, ****p≤0.0001.

To further explore the effect of pitavastatin on bHLH gene expression, we analyzed the expression of E47 and the 4 HLH Id proteins. Somewhat surprisingly, 10 μM pitavastatin treatment for 48 hours did not alter the mRNA expression patterns of E47 or ID1-3 in a consistent manner across the 3 cell lines (Figure 5) and ID4 levels were below the limit of detection. Because the bHLH protein Dec-1/bHLHe40/STRA13/SHARP2 has been linked to increased gemcitabine sensitivity and improved prognosis in PDA [29], we examined Dec-1 expression in response to pitavastatin. Strikingly, pitavastatin induced significant upregulation of Dec-1 mRNA in all PDA lines. Moreover, all 9 statins triggered a significant increase in Dec-1 expression in PANC1 cells (Figure 5). In contrast, RT-qPCR analysis of mRNA expression for the bHLH proteins Atoh8, Slug, and c-myc did not yield consistent results across all cell lines, and Twist and HES-1 could only be detected in 779e cells (Supplementary Figure 9). The levels of the bHLH proteins HEB (TCF12) and PTF1a were below the limit of detection in all cell lines, both before and following statin treatment. Given the role of some bHLH proteins in epithelial to mesenchymal transition (EMT) we also examined the effects of pitavastatin on E-cadherin or vimentin, finding that drug treatment did not uniformly alter their expression across all lines (Supplementary Figure 9).

### Pitavastatin reduces Rb phosphorylation and represses E2F-regulated proliferation of target genes

To further investigate signaling pathways regulated by pitavastatin, we considered that downstream effectors of Kras might be altered. Activation of Kras leads to phosphorylation of its effector protein ERK. Consistent with activated Kras, phospho-ERK was readily detectable in all 3 lines, including BxPC3 which has wild-type, not mutant Kras. Pitavastatin treatment, however, did not induce uniform changes in phospho-ERK, relative to total ERK protein, across the 3 lines, suggesting that pitavastatin employed alternate pathways to inhibit PDA cell growth (Supplementary Figure 10).

**Figure 6:**
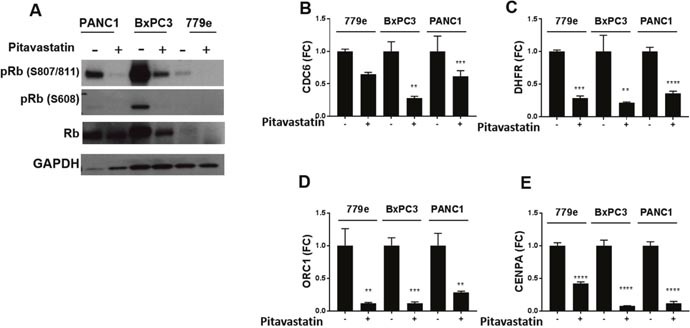
Pitavastatin represses Rb phosphorylation and E2F target gene expression PANC1, BxPC3, and 779e cells +/−10 μM pitavastatin treatment were harvested for protein and RNA expression. **(A)** Western blot analysis of Rb phosphorylation status at Ser807/811 and Ser608, total Rb, and GAPDH control. RT-qPCR of **(B)** CDC6, **(C)** DHFR, **(D)** ORC1, and **(E)** CENPA. Data are represented as mean +/−SD, *p≤0.05, **p≤0.01, ***p≤0.001, ****p≤0.0001.

In PDA, diminished expression of the CDK inhibitors p21 and p16 enables repressive phosphorylation of the Rb tumor suppressor by CDKs [4]. The significant increase in p21 signal and protein in response to statins prompted us to investigate whether pitavastatin blocked Rb phosphorylation at CDK4/6 regulated serines 807/811 and 608. Strikingly, pitavastatin treatment provoked significant inhibition of Rb phosphorylation at 807/811 in all lines and attenuated Rb phosphorylation at serine 608 was also readily apparent in BxPC3 and PANC1 cells (Figure 6A). Consistent with a previous report that statins inhibited Rb phosphorylation but also lowered total Rb in some cells [30], we also observed a reduction in total Rb protein in BxPC3 and 779e, but not PANC1 cells.

Rb tumor suppressor activity occurs through direct repression of a set of E2F factors that promote expression of proliferation associated genes. E2F factors govern numerous cell cycle genes involved in the G1/S checkpoint, including components of the pre-replication complex (PRC) that license initiation of DNA synthesis. Importantly, the pre-replication complex cannot function in the absence of the constituent ORC1, that recognizes origins of replication, or the ATPase, CDC6. Consistent with Rb activation and E2F inhibition, pitavastatin significantly downregulated ORC1 and CDC6 expression in all lines. Moreover, pitavastatin also suppressed the expression of the E2F target CENPA, a centromere factor required for replication, as well as DHRF, a metabolic enzyme required for nucleotide synthesis (Figure 6B-6D). Together the data suggest that pitavastatin recruits Rb to block the G1/S transition in PDA cells by upregulating the CDK inhibitor p21. Consistent with the notion that CDK inhibition is critical to suppressing PDA cell growth, the CDK inhibitor Palbociclib (PD-0332991) also repressed proliferation of all 3 cell lines (Supplementary Figure 11).

### Pitavastatin induces low level expression of acinar enzymes and the bHLH protein MIST1

**Figure 7:**
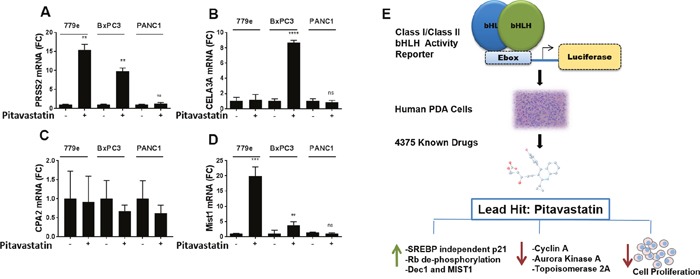
Pitavastatin induction of acinar differentiation genes BxPC3, PANC1 and 779e cells treated with 10 μM pitavastatin for 48hr were harvested for RT-qPCR analysis of acinar differentiation markers: **(A)** PRSS2, **(B)** CELA3A, **(C)** CPA2 and **(D)** MIST1. Data are represented as mean +/−SD, *p≤0.05, **p≤0.01, ***p≤0.001, ****p≤0.0001. **(E)** A graphic model describing assay development, drug discovery and mechanistic studies with statins.

During the progression of PDA, tumorigenic cells undergo a loss of acinar differentiation and take on a stem-like phenotype [5–7]. We recently established that increasing the expression of E47 in highly tumorigenic PDA cells induces robust acinar reprogramming in addition to stable growth arrest *in vitro* and *in vivo* [17]. Pitavastatin induced a 15 fold increase in trypsinogen 2 message in 779e and a 10 fold increase in BxPC3 (Figure 7), although this is far below the level induced by E47 overexpression (Supplementary Figure 11 and ref [17]). A second acinar enzyme, elastase 3A (CELA3A), was induced 8 fold in BxPC3 only, and carboxypeptidase A2 (CPA2) was not significantly induced in any cell lines (Figure 7). Pitavastatin also elicited expression of acinar maturation bHLH protein MIST1 in 779e (19.9 fold, p=4.5e-4) and in BxPC3 cells (3.8 fold, p=3.2e-4) (Figure 7) as we had previously observed in E47 overexpressing cells [17]. In contrast, PD-0332991 did not induce trypsinogen expression in any of the lines (Supplementary Figure 11). Thus, CDK and growth inhibition are not sufficient to induce acinar enzymes, suggesting that pitavastatin upregulates trypsinogen by CDK independent mechanism.

In summary, a screen for inducers of Class I/ClassII bHLH factors identified pitavastatin as a significant hit. We show that pitavastatin induces transient cell cycle arrest associated with upregulation of p21, decreased Rb phosphorylation, and reduced pro-proliferation target genes across 3 genomically diverse PDA lines (Figure 7E).

## DISCUSSION

The 5-year survival rate for PDA remains in the single digits, suggesting that novel therapeutic approaches are needed for this recalcitrant cancer. Based on our recent finding that the Class I bHLH E47 elicits robust acinar reprogramming and stable growth arrest in PDA cells, we developed a high throughput screening assay for ClassI/II bHLH activity. The assay was designed to cast a wide net in order to capture compounds that promote bHLH function by a variety of mechanisms, e.g. increased bHLH expression, nuclear localization of bHLH proteins, or repression of ID factor expression or function. Though our reporter was selectively designed to detect Class I/II bHLH activity, the assay design does not exclude the possibility of identifying a drug that increases activity of a pro-growth bHLH protein, e.g. the bHLH-Zip protein c-myc. Such hits, however would later be eliminated in secondary studies.

Screens to identify pharmacologic agents for cancer often use cytotoxicity as a readout but their binary nature can preclude identification of drugs which are weakly active and could be effective at an alternate dose. Here we developed a tunable assay with an incremental readout of bHLH activity. The bHLH reporter assay is also rapid; it is strongly induced within 48 hours of tamoxifen treatment in positive control cells expressing E47^MER^ and this corresponds with profound changes in molecular markers of cell cycle arrest, including increased p21. Importantly, the molecular changes occur prior to a statistical change in cell number [17]. Therefore our approach offers an advantage over a proliferation based assay requiring longer incubations, media changes, and repeated compound treatment. While the PANC1/E47 line served as a potent positive control for the assay, these cells were not employed in the final screening platform. [19, 21].

In a screen of 4375 known pharmaceuticals, important parameters of assay performance were met. The hits were evenly distributed across the plates, the majority of compounds overlapped with DMSO negative controls in a normal distribution, and several hit compounds were identified from 2 or 3 libraries.

Statin compounds were prominent among the hits in the screen. Statins are cholesterol lowering drugs that inhibit the catalytic activity of HMG-CoA reductase, and are among the most therapeutically effective pharmaceuticals created. The potential antineoplastic properties of statins have received significant attention in recent years, although the efficacy of statins as single agents in PDA remains under debate [31]. A large clinical study found reduced PDA risk in men with prolonged statin use [32] and others suggest that statins are beneficial in patients with low grade or resectable PDA [33, 34]. In the murine model of PDA that harbors activated Kras and mutant p53 (the KPC mouse), simvastatin and lovastatin have been shown to slow pathogenesis [35, 36]. Interestingly, a large meta-analysis of 16 placebo and standard care–controlled statin trials with 113,800 participants reported that statins are protective against pancreatitis, which is a significant risk factor for PDA [37].

Here we investigated pitavastatin treatment in 3 PDA lines, PANC1, BxPC3 and a more differentiated, low passage line recently derived from a primary tumor, 779e. In all 3 cell lines pitavastatin slowed growth which was quickly reversed in PANC1 and BxPC3 cells when drug was removed. Growth reversal was not apparent in 779e cells during the length of the experiment but these cells may simply take longer to recover as their proliferation rate is low in general.

Several biologic mechanisms by which statins may affect tumor growth have been proposed. Statins have been reported to stimulate anticancer immune surveillance [38]. Our studies however, utilized isolated epithelial PDA cell cultures, indicating that statins can also diminish PDA cell proliferation *in vitro* through immune independent mechanisms. Statins inhibit mevalonate biosynthesis that then represses the formation of downstream lipid isoprenoid intermediates, such as farnesyl PPi (FPP) and geranylgeranyl PPi (GGPP). Isoprenoid lipid moieties are added to Ras through posttranslational modification (prenylation), and are required to anchor Ras to the cell membrane [39]. The BxPC3 line does not harbor a Kras mutation, but because wild-type Ras signaling can also be activated in cancer, we queried the effects on Ras signaling via phosphorylation of the effector, ERK1/2. Pitavastatin did not induce uniform alterations in phospho-ERK across the 3 lines, suggesting that statins can inhibit PDA cell proliferation through alternate cues.

A key aspect of our findings is the pronounced and dose responsive upregulation of the CDK inhibitor p21 in all cell lines tested. Consistent with our results, atorvastatin was found to upregulate p21 expression in the KPC model of PDA [36]. p21 is generally a p53 target gene yet none of the cell lines employed in these studies express wild-type p53 and recent ChIP assays showed that mutant forms of p53 found in PDA cells cannot bind to the p21 promoter [40]. We previously showed that E47 binds directly to the p21 promoter which contains four E-boxes [17]. In addition to E47, the bHLH protein MIST1 has also been shown to activate p21 [41]. The bHLH-ZIP transcription factor SREBP1, the target responsible for statins’ cholesterol lowering effects, induced p21 in osteosarcoma and HepG2 cells [42], prompting us to investigate whether statins utilized SREBP1 to induce p21 in PDA cells. We provide 2 lines of evidence however that SREBP1 is not required for pitavastatin induced p21 expression or for cell cycle exit in PDA cells. We cannot rule out the possibility however, that the low levels of cleaved (active) SREBP1 remaining after cholesterol treatment of cells and the 23% of SREBP1 remaining after siRNA mediated knockdown are sufficient to induce p21.

Because statins robustly upregulated p21, we considered that this would restore Rb activity by blocking CDK mediated Rb phosphorylation. Consistent with this model, pitavastatin treated PDA cells were refractory to Rb phosphorylation at CDK targeted serines 807/811 and 608. Also consistent with Rb activation was the finding that multiple direct target genes of the canonical proliferation-associated E2Fs (1, 2, 3a) were repressed by pitavastatin in all PDA lines. In particular, components of the pre-replication complex required for the G1/S transition were significantly blunted by pitavastatin treatment. Not all E2Fs however are pro-proliferation; e.g. E2F4 and E2F7 directly repress proliferation targets Thus statins, possibly through p21, may additionally activate repressive E2Fs [43, 44].

As observed in statin treated prostate cancer cells [30], alleviation of Rb phosphorylation was accompanied by reduced total Rb protein levels in 2 of the 3 lines examined here. Interestingly, PDA cells treated with the CDK4/6 Inhibitor Palbociclib (PD-0332991) activate Rb as expected and also exhibit lower total Rb protein [45] as do cells with G1 arrest induced by other means [46].

Statins did not significantly alter E47 and ID factors at the mRNA level. Due to the complexity of bHLH regulation however, future studies will be required to probe bHLH and ID factors at the level of protein expression, phosphorylation status, and subcellular localization to identify a possible role for these and other bHLH proteins in statin induced p21 induction and growth arrest. Intriguingly, of 12 bHLH/ID factors examined at the RNA levels, only the hypoxia inducible bHLH protein Dec-1 was significantly induced in all lines treated with pitavastatin. Moreover, in PANC1 cells, we show that all 9 statins uniformly elevated Dec-1 expression. Dec-1 plays a role in circadian rhythm [47] and in p53/p21 dependent senescence [48]. A recent study suggested that Dec-1 is a novel prognostic marker in pancreatic cancer, associated with increased gemcitabine chemosensitivity [29]. Thus, it might be expected that statins (with potentially upregulated Dec-1) could be effective as adjuvant therapy with gemcitabine. In a retrospective study, statin use was found to be protective in patients independent of gemcitabine treatment [49]. Interestingly, the statin benefit was independent of cholesterol level, consistent with our finding that statin induced p21 expression is cholesterol and SREBP1 independent. In a phase II placebo controlled clinical trial however, simvastatin did not provide clinical benefit to PDA patients treated with gemcitabine [50]. Thus the effectiveness of a combined statin/gemcitabine therapeutic strategy in PDA remains controversial.

We previously found that restoring bHLH signaling in PDA cells promoted robust acinar reprogramming of the cells, revealing a previously unrecognized plasticity in PDA [17]. Reprogramming the differentiation state of cancer is a therapeutic strategy that has worked extremely well in Acute Promyelocytic Leukemia (APL), changing this once lethal cancer to one that is fully treatable [51]. In the studies presented here, we utilized the established lines PANC-1 and BxPC3 as well as a line recently derived from a patient tumor, 779e. Based upon baseline expression of the acinar enzyme trypsin 2, 779e is the most differentiated line, followed by BxPC3, and both are significantly more differentiated than PANC-1 cells. Pitavastatin induced expression of the acinar enzyme trypsinogen in BxPC3 and 779e, although at levels far below that induced by E47 overexpression (17 and Figure 7). The data suggest however that the more differentiated lines at baseline were more sensitive to statin induced upregulation of acinar genes. Overall, however, either higher levels of bHLH activity are required for global acinar reprogramming or a specific bHLH protein, not induced by pitavastatin, plays a pivotal role in acinar cell fate. The acinar bHLH protein PTF1a was not consistently detectable in the cell lines although we previously showed that high levels of E47 activity can compensate for the absence of PTF1a [17]. The CDK inhibitor PD-0332991 did not induce trypsinogen expression in our studies although it repressed PDA proliferation (as has been reported by others) [45]. Therefore, slowing growth alone does not promote acinar enzyme expression.

Palbociclib is currently in clinical trials as an adjuvant therapy for PDA, although preclinical studies have identified potential disadvantages of the drug regarding PDA invasiveness [45]. Given that neither statins nor Palbociclib appear to be effective as single agents in PDA, re-activating Rb alone may not be sufficient to significantly alter clinical outcome in PDA. The c-myc gene is amplified in nearly half of PDA tumors [52] and many studies have shown the critical role that c-myc plays in PDA pathogenesis [53]. We show here that pitavastatin did not uniformly lower c-myc mRNA expression across 3 PDA lines. Nor did statins uniformly downregulate phosphorylation of the Ras effector ERK1/2 which promotes myc protein stabilization in PDA [54]. Thus, an effective strategy for PDA may require combined Rb activation and c-myc inhibition.

In conclusion, we have developed a novel screening platform for bHLH activity and established its value as a tool for drug discovery in pancreatic cancer. The data also provide mechanistic insights into the role of statins in PDA. Concordant data across three genomically diverse PDA lines suggest that statins modulate oncogenic cues through bHLH signaling, p21, CDK inhibition, and Rb activation to regulate E2F target genes and proliferation. It will now be of interest to probe additional hits from the screen, individually, and for potential synergy with statins, to promote pharmacologic induction of high level bHLH signaling, stable cell cycle exit, and robust acinar reprogramming.

## MATERIALS AND METHODS

### Cell culture

Human PDA cell lines PANC1 (CRL-1469) and BxPC3 (CRL-1687) were maintained in RPMI-1640 media containing 10% fetal bovine serum and penicillin/streptomycin in 5% CO2 at 37C. Cell line identity was verified by DNA fingerprinting. A PANC1 derivative cell line stably expressing an inducible form of E47 was previously described [18, 19]. Briefly, cells were infected with a retroviral vector expressing E47 fused to a modified estrogen receptor (MER), herein named PANC1/E47 779e is a patient derived cell line established by A. M. Lowy, and these cells were cultured in DMEM supplemented with 12% fetal bovine serum, fungizone, penicillin/streptomycin, sodium pyruvate, MEM essential amino acids, L-glutamine. Cholesterol (EMD Millipore) was dissolved in hot ethanol and sterile filtered before use in cell culture media at 10μg/mL. Drug treatments were performed for 48-72 hours with vehicle, 1 uM PD-0332991, or 2, 5 or 10 uM statins.

### Cell transduction

The bHLH reporter expresses luciferase driven by 4 multimers of 3 unique bHLH binding elements ‘E-boxes’ (Supplemental Figure 1) [20]. The SREBP1 reporter and SREBP1a expression plasmids were a kind gift from Dr. Timothy Osborne, as previously described [27, 28]. DNA plasmids were transfected with Viafect per manufacturer's instructions. Luciferase activity was measured using Brightglo (Promega) per manufacturer's instructions. Gene knockdown studies utilized pools of 4 Dharmacon siRNAs for SREBP1 (gene name SREBF1): J-006891:05-08. NS1 and NS2 were each pools of non-specific siRNAs. Briefly 2ul of 0.5uM pooled siRNA was spotted into each well of a 96 well plate, then 20ul of RNAiMAX/OptiMEM mixture was added to each well and incubated for 30 min. Finally, 80ul of cells at density 100,000 cells/ml was added to each well and incubated for 72 hours in the presence or absence of drug.

### Assay design

On Day 1, PANC1 cells were transfected with the bHLH reporter using 6uL ViaFect transfection reagent (Promega) per 1 ug of DNA plasmid in wells of a 6-well plate. On Day 2 transfected cells were resuspended, pooled, and transferred to tissue culture treated 96 or 384 well white plates (Greiner) using a Multi-Drop Combi (Thermo Scientific). On Day 3 compounds were dispensed using Labcyte Echo 555 Acoustic Pipetter. On Day 5 Promega Bright-Glo luciferase substrate was added using a Multi-Drop Combi and the luciferase activity was measured using BMG labtech PHERAstar. Results were analyzed using Genedata Analyst.

### Compound libraries

The screen consisted of 5 libraries: Prestwick chemical library (1280 compounds), Library of Pharmacologically Active Compounds (LOPAC)(1280 compounds), FDA/International drug library (1280 compounds), NIH clinical collection (446 compounds), and NCI oncology library (89 compounds). In total, 4,375 total drugs were tested. For confirmatory studies, all statins were obtained as dry powder from Selleckchem with the exception of cerivastatin which was purchased from Sigma. The purity of all compounds was verified by LC-MS analysis with all found to be >95% pure.

### Statistical analysis

The screen was analyzed using Gene data Analyst. Z’ factor, a measure of drug assay reliability, uses the mean and standard deviation of positive and negative controls to calculate of accuracy. Z-factor was calculated using the mean and standard deviations of the positive and negative controls. Z-score were calculated for each hit as the raw score minus the mean score of the population normalized to the standard deviation of the population.

### Quantitative PCR

RNA was extracted with an RNeasy Mini Kit (Qiagen) and reverse transcribed with qScript cDNA Supermix (Quanta). Real-time qPCR was performed using the LightCycler 480 II system with SYBR Green I (Roche), and gene expression was normalized to 18S rRNA. Primer sequences are provided in Supplemental Table 1. P values are designated as: *p≤0.05, **p≤0.01, ***p≤0.001, ****p≤0.0001.

### Immunoblotting

Whole-cell extracts were prepared by incubation in RIPA buffer containing protease inhibitors (Calbiochem). Protein (20 ug) was separated on 4% to 20% Longlife gels (LifeGels) and transferred to Immobilon-P membrane (Millipore). Membranes were blotted with antibodies to p21CIP1/WAF1, Rb, p807/811-Rb, p608-Rb, phosph-T202/Y204 ERK, ERK1/2, and Vinculin (Cell Signaling) SREBP1A (Santa Cruz) GAPDH (GeneTex), washed and incubated with HRP-conjugated goat secondary antibodies (Santa Cruz) and imaged with film, which was processed by a M35A-X-OMAT (Kodak).

### Immunofluorescence

Cultured cells were fixed in 4% paraformaldehyde (USB Corp), permeabilized with 0.3% Triton X-100, and blocked with 5% donkey serum (Jackson ImmunoResearch Laboratories) and 1% bovine serum albumin (Sigma) in PBS. Primary antibody: mouse monoclonal anti-Ki67 (BD Biosciences 550609, 1:100). Secondary antibody: Rhodamine red donkey anti-mouse (Jackson ImmunoResearch Laboratories). Nuclei were counterstained with DAPI (4′, 6-diamidino-2-phenylindole dihydrochloride; AppliChem). Samples were mounted in Vectashield (Vector Labs). Digital images were acquired with fluorescence microscope equipped with a digital camera (Nikon).

## SUPPLEMENTARY FIGURES AND TABLE


