# Supplementary material for: A screen for inducers of bHLH activity identifies pitavastatin as a regulator of p21, Rb phosphorylation and E2F target gene expression in pancreatic cancer
Source: Oncotarget. 2017 Jun 21;8(32):53154–67. doi: 10.18632/oncotarget.18587 (PMC5581100; doi:10.18632/oncotarget.18587)
Supplement: Supplementary file 1 [file oncotarget-08-53154-s001.pdf]

A screen for inducers of bHLH activity identifies pitavastatin as a regulator of p21, Rb phosphorylation and E2F target gene expression in pancreatic cancer

SUPPLEMENTARY FIGURES AND TABLE

AGATCT**AGAACACCTGCAGCAGCTGGCAGGGAT**
CTGGTCATGTGGCAAGGCTATTTGGGATCTAGAA
CACCTGCAGCAGCTGGCAGGGATCTGGTCATGTG
GCAAGGCTATTTGGGATCTAGAACACCTGCAGCA
GCTGGCAGGGATCTGGTCATGTGGCAAGGCTATT
TGGGATCTAGAACACCTGCAGCAGCTGGCAGGG
ATCTGGTCATGTGGCAAGGCTATTTGGGATCCACT
AGTTCTAGAGGATCCGCCGCGGCCGC*CTTTATAA*
GGCGGCGGGGGTGGTGGCCCGGGCCGCGTTGCG
CTCCCGCCACTCCGCGCCCGCTATCCTGGCTCCGTG
*CTCCCACGCGCTTGTGCCTGGACGGACC*AAGCTT
GGAATTCCTTTGTGTTACATTCTTGAATGTCGCTCG
CAGTGACATTAGCATTCCGGTACTGTTGGTAAA*AT*
G

Supplementary Figure 1: E-box reporter sequence. The reporter sequence beginning with the BglII/NotI insert containing 4 copies of ligated XbaI fragments, each containing 3 E-boxes (μE5/μE2/μE3) from the mouse immunoglobulin heavy chain gene (in boldface), and ending with ATG from luciferase cassette.

A

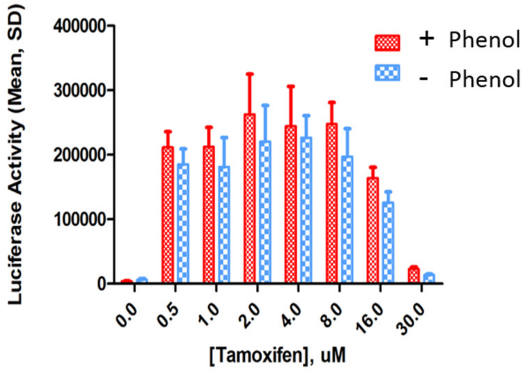

B

	RPMI only	No Tamoxifen			Tamoxifen 4uM		
Cell No.	0	5,000	7,500	10,000	5,000	7,500	10,000
A	280	3,000	5,120	14,600	400,520	619,960	794,440
B	280	3,840	5,040	20,040	496,920	513,480	1,346,480
C	280	5,200	8,720	14,600	464,600	773,040	1,191,200
D	320	3,520	4,640	7,120	298,280	594,640	821,160
E	280	3,440	6,840	7,640	352,920	430,360	1,046,280
F	280	3,840	7,280	9,040	458,800	651,840	1,056,840
G	320	2,840	6,680	9,640	668,200	519,520	1,192,080
H	360	3,920	6,560	9,600	295,880	638,720	1,044,000
I	320	5,200	9,040	10,120	528,760	515,040	908,600
J	440	3,680	5,360	9,840	560,920	664,920	965,840
K	280	3,040	8,040	8,800	611,800	520,760	1,100,400
L	320	5,240	4,840	9,560	515,320	730,120	792,040
M	400	3,520	11,120	8,920	444,880	786,680	1,247,360
N	280	5,560	6,280	8,600	287,240	602,080	780,440
O	280	10,800	7,600	14,960	550,080	732,840	1,034,280
P	320	2,400	8,080	16,120	634,560	704,320	378,040
Mean	0	4,000	6,638	10,885	472,790	624,580	980,903
SD	48	1,971	1,787	3,670	121,038	105,010	235,181
Z-factor		0.213	0.482	0.261			

Supplementary Figure 2: Assay optimization. (A) Dose responsive luciferase activity (0 to 30 µM tamoxifen for 48 hours) was determined in PANC1/E47^{MER} cells transfected with the E-box-luc reporter. The possibility of phenol red interference with luciferase signal was also investigated. **(B)** To optimize cell density in a 384 well format, a Z’ was calculated for PANC1/E47^{MER} cells expressing the luciferase reporter, plated at 5000 to 10,000 cells per well +/- 4uM tamoxifen.

	P1E47 WT	P1 DMSO	P1 DMSO	P1 DMSO	P1 DMSO	P1 DMSO	P1 DMSO	P1 DMSO	P1 DMSO	P1 DMSO	P1 DMSO	P1 DMSO	P1 DMSO	P1 DMSO	P1 DMSO	P1 DMSO	P1 DMSO	P1 DMSO	P1 DMSO	P1 DMSO	P1 DMSO	P1 DMSO	P1 DMSO
	1	2	3	4	5	6	7	8	9	10	11	12	13	14	15	16	17	18	19	20	21	22	23
A	599395	3910	2815	3540	3630	2535	2320	3420	2325	2340	2650	1825	2645	2110	2620	2715	2275	2355	2185	2590	2535	2895	2185
B	638920	3135	2090	1885	2605	2405	2875	2370	2345	1920	2555	2355	2575	1995	2400	2275	2285	1970	2265	2035	2020	1745	2005
C	524760	4185	2445	2925	3345	2435	2650	2195	1935	2135	2490	1810	1935	2215	2200	2045	2595	1685	2665	2890	2740	2345	2405
D	435370	2925	3105	2400	3140	2325	2425	2780	1720	2425	2000	2470	2465	2620	2225	2550	2210	2795	1900	2100	1720	2065	2210
E	515550	2795	3740	3255	2800	2815	2680	2105	2520	2685	3570	2795	2130	2070	2505	2270	2225	2005	2620	2410	2135	2640	2645
F	713645	2965	2670	3450	3200	2880	2180	2945	2320	3005	1810	2095	2030	2725	2365	3395	2700	1845	2150	2045	2185	1685	1625
G	604005	3890	3750	2860	3165	3420	3270	2300	2000	2185	2255	1960	2710	2755	2580	1960	3480	2105	2115	1805	2620	2510	2110
H	558420	4045	2210	3480	3115	2395	2980	2885	2500	2905	2550	2165	2465	2775	2965	2355	2845	1960	3015	2205	2170	2075	2285
I	602790	4415	3270	2645	3070	3215	2535	2410	2010	2385	1755	2380	2420	2595	2855	2680	2425	2440	2255	2585	2205	2065	1810
J	556825	4150	3380	3000	2735	2910	2495	3120	2320	2880	2115	2015	2405	2765	2755	2525	2685	2545	2255	2130	2590	1945	2455
K	669390	4435	3410	2465	2875	2490	2470	3230	2570	2440	2525	2100	2220	2470	2170	2425	2645	3355	2270	2495	2030	2150	1575
L	700325	4450	2740	3225	2145	3120	2200	2170	3545	2075	1950	2175	2050	2685	2790	2475	3100	2190	2790	2350	2430	2510	2465
M	637945	3985	3255	2880	2550	3035	2875	2335	2460	2705	1880	2060	2360	2210	2525	1945	3265	2480	2795	2450	2405	1920	1980
N	673125	3075	3265	2750	2020	2735	2395	3710	1920	2665	2175	1740	2940	3120	2730	2000	3170	1790	1595	2170	2375	2285	2300
O	743540	3325	3700	2530	3395	2390	3445	2755	2255	3290	2340	1835	2315	2530	1960	2245	3255	3880	2450	2395	2580	2550	2390
P	708970	2665	2445	1855	2675	1770	1865	1815	1705	2085	1580	2460	2010	1960	1845	2050	2475	2130	2130	2375	1890	2485	1555

Supplementary Figure 3: Z factor determination. Parental PANC1 cells (P1) expressing E-box-luc were plated in 352 wells of a 384 well plate and treated with DMSO (negative control). PANC1/E47^{MER} cells (P1/E47) expressing E-box-luc were plated in 16 wells of the plate and treated with 4 µM tamoxifen as a positive control. Luciferase was assayed following 48 hours of incubation.

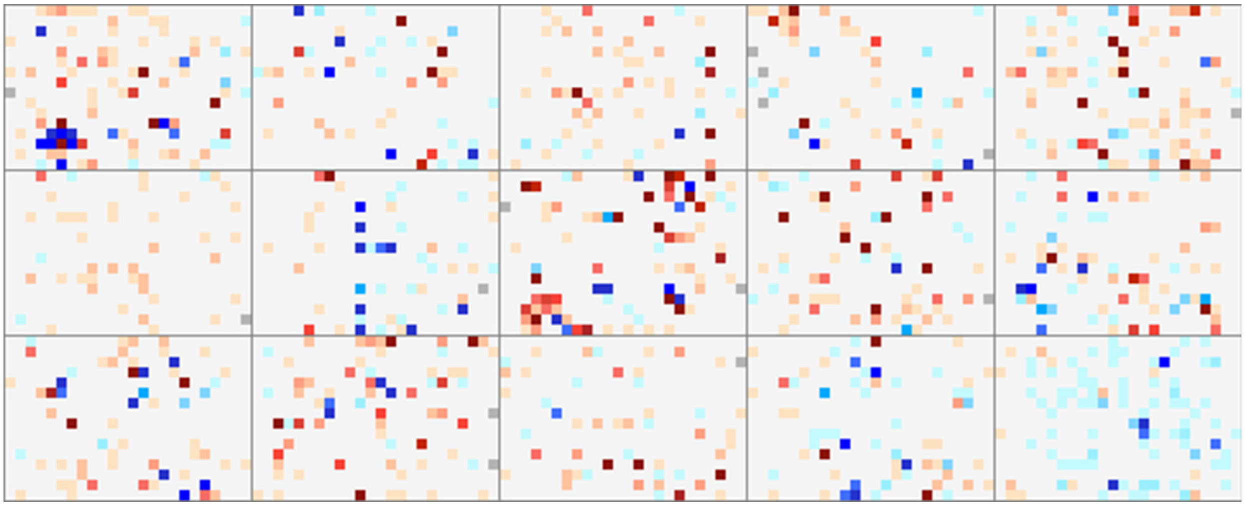

Supplementary Figure 4: Plate distribution of hits. Hits (red pixels) from a screen of 4375 pharmaceuticals were distributed across 14 of 15 plates. The highest Z scores are in dark red (29.52) and the lowest are in dark blue (-8.77).

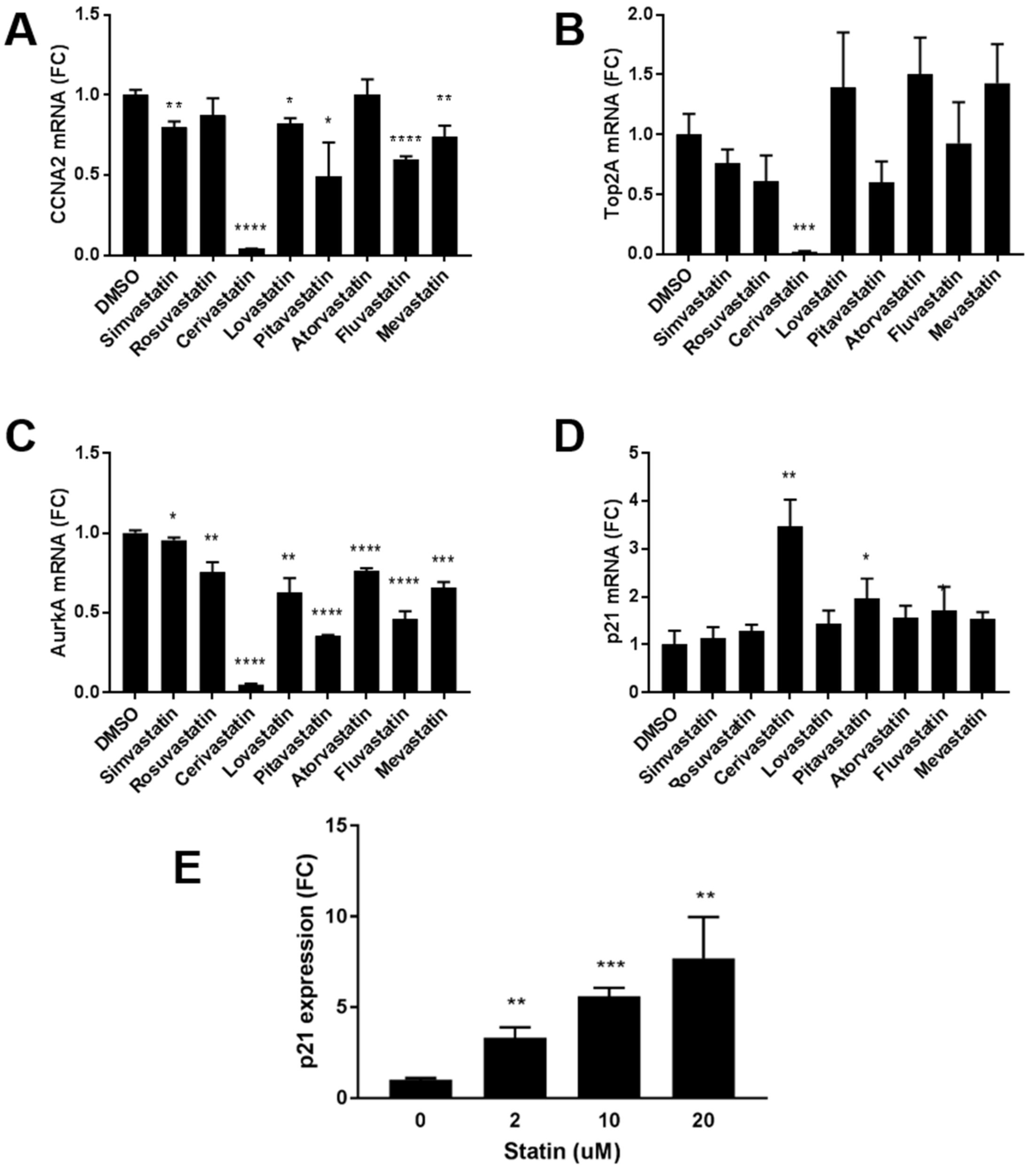

Supplementary Figure 5: Statins regulate expression of cell cycle related genes at 5µM. PANC1 cells were treated with 5 µM of each of the 9 statins for 48 hours. RT-qPCR data is shown for: **(A)** CCNA2, **(B)** TOP2A, **(C)** AURKA and **(D)** p21 as fold change (FC) over DMSO treated cells. **(E)** P21 induction is dose responsive. Data are represented as mean +/-SD, *$p \leq 0.05$, **$p \leq 0.01$, ***$p \leq 0.001$, ****$p \leq 0.0001$.

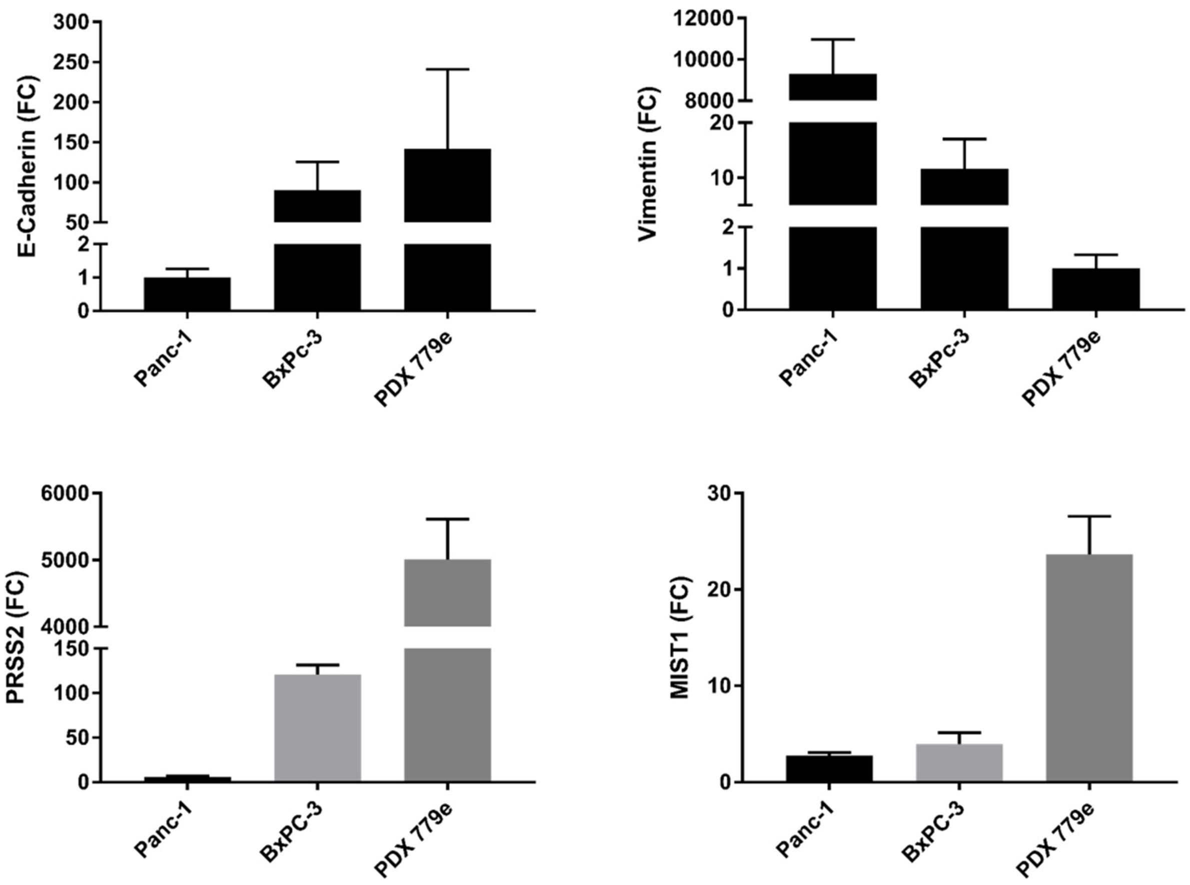

Supplementary Figure 6: Cell line characterization. The baseline mRNA expression levels of E-cadherin, vimentin, Trypsin 2 (PRSS2) and MIST1 in parental PANC1, BxPC3 and 779e cells as measured by PCR. All values were first normalized to 18s and then one cell line was set at 1 for each gene. Data are represented as mean +/-SD.

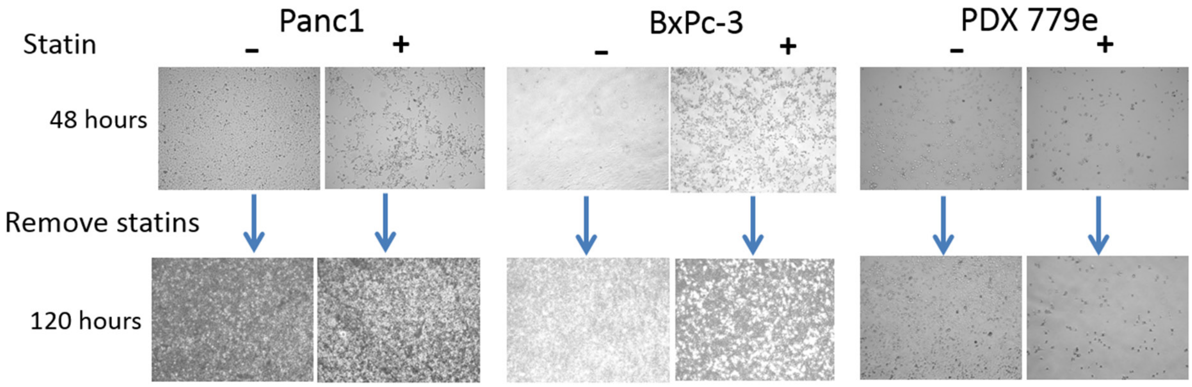

Supplementary Figure 7: Proliferation following removal of pitavastatin. In a "washout" experiment, cells were treated with pitavastatin for 48 hours after which they were washed and cultured in basal media without drug for 72 hours. Bright field images are 50x original.

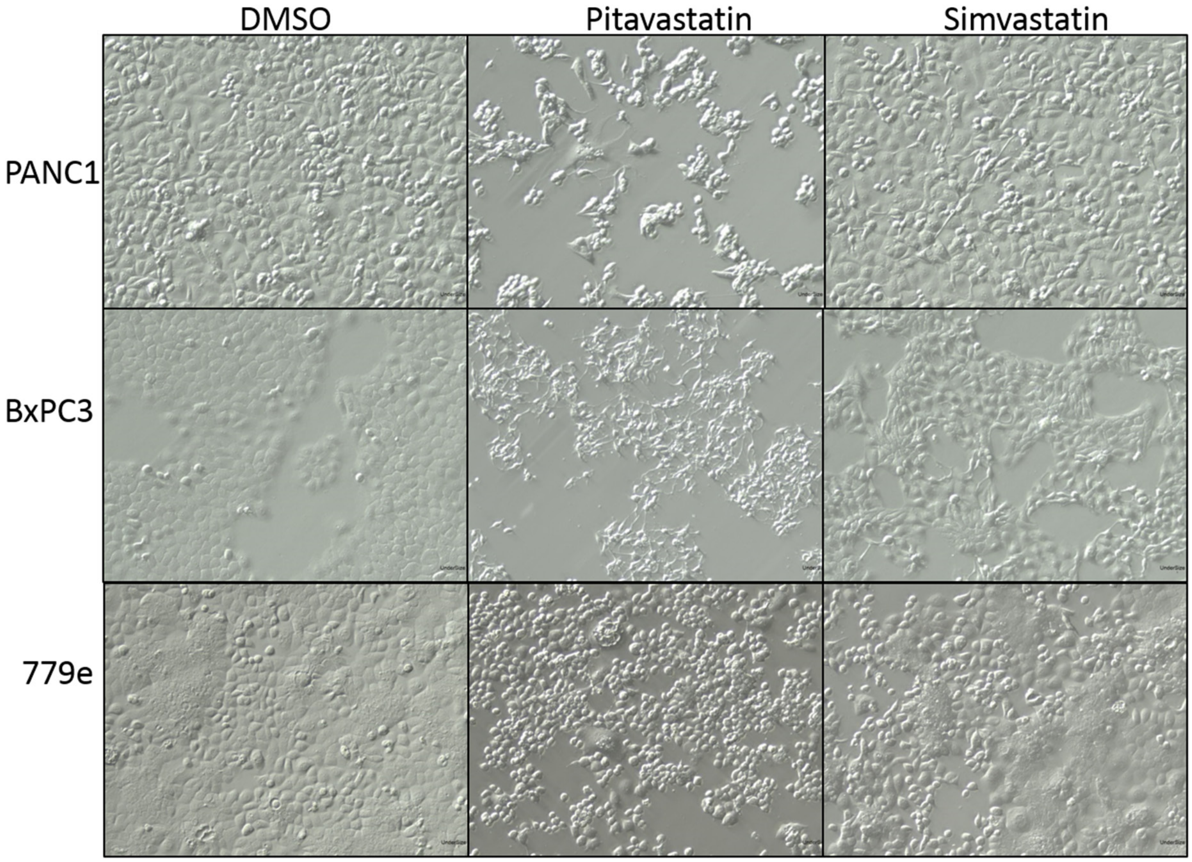

Supplementary Figure 8: Cytoskeletal alterations in simvastatin versus pitavastatin treated PDA cells. Treatment of BxPC3, PANC1 and 779e cells 10 µM simvastatin for 48 hours does not induce cytoskeletal alterations compared with 10 µM pitavastatin. Bright field images are 100X original.

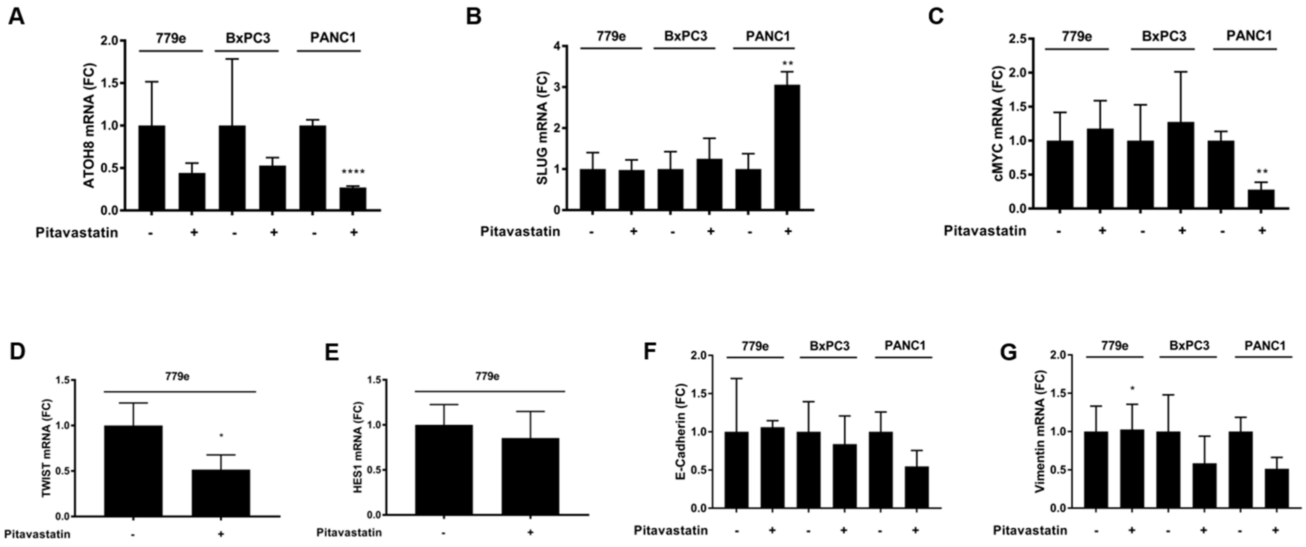

Supplementary Figure 9: Pitavastatin induced expression of bHLH factors and EMT markers. PANC1, BxPC3, and 779e cells were treated with 10 µM pitavastatin for 48 hours and mRNA expression was measured by PCR for **(A)** Atoh 8, **(B)** SLUG, **(C)** cmyc. Only 779e cells exhibited expression of **(D)** Twist and **(E)** Hes1. Pitavastatin did not alter expression of **(F)** E-cadherin or **(G)** vimentin in the 3 lines.

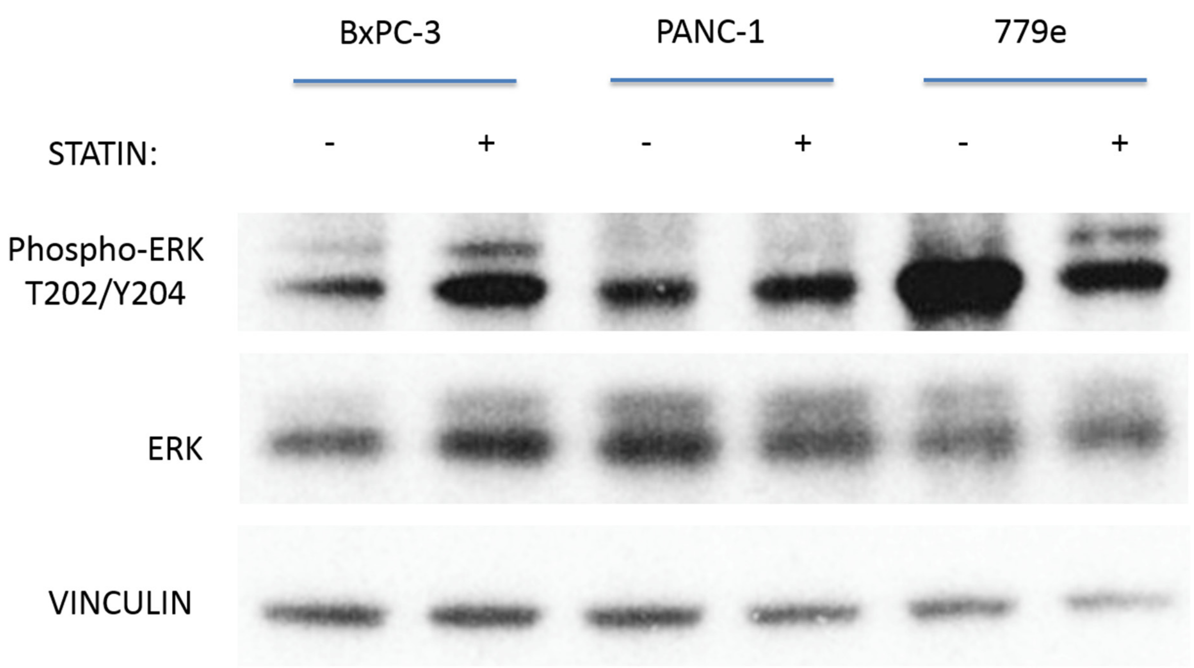

Supplementary Figure 10: Pitavastatin effects on ERK1/2 phosphorylation. PANC1, BxPC3, and 779e cells were treated with 10 μM pitavastatin for 48 hours after which they were harvested for Western blots of Phospho-ERK1/2 (T202/Y204), total ERK1/2 protein and vinculin.

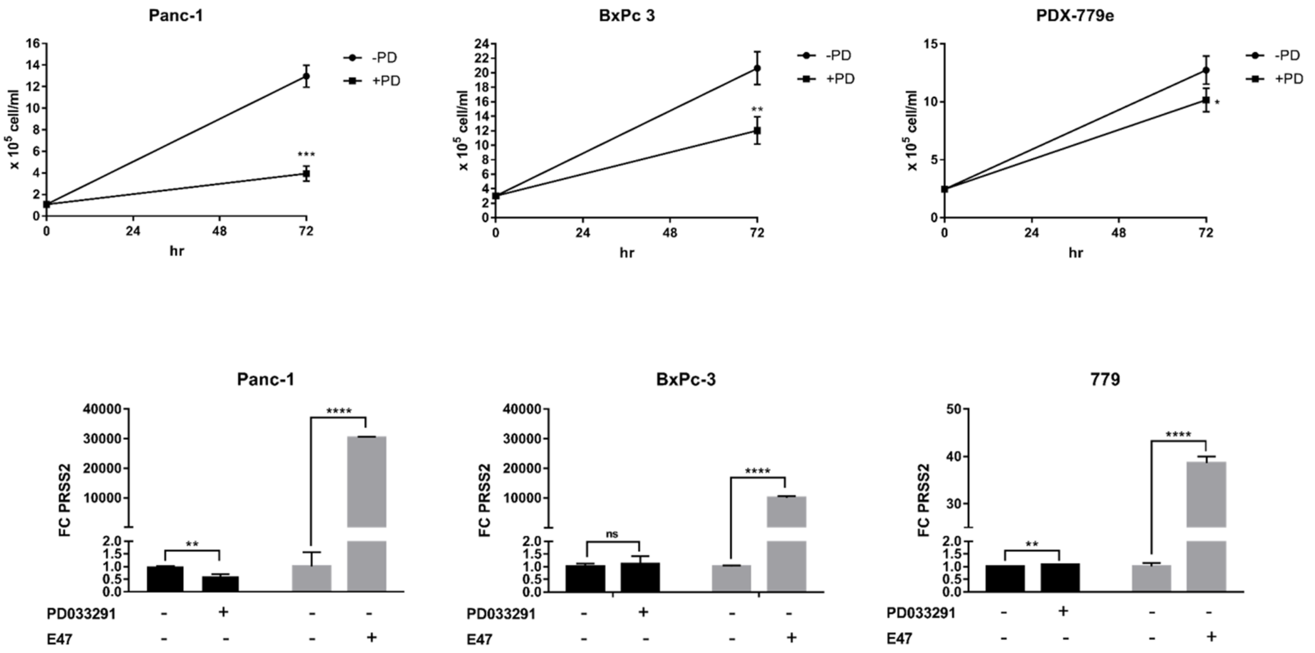

Supplementary Figure 11: CDK Inhibition slows cell growth but does not induce trypsinogen expression. PANC1, BxPC3, and 779e cells were treated with 1uM PD-0332991 for 72 hours. Each line exhibited slowed growth, but trypsinogen was not induced in any of the lines. PANC1/E47^{MER} cells treated with 4μM tamoxifen served as a positive control for trypsinogen expression. *p≤0.05, **p≤0.01, ***p≤0.001, ****p≤0.0001.

Supplementary Table 1: PCR primers

Gene	Forward	Reverse
18S	GATATGCTCATGTGGTGTTG	AATCTTCTTCAGTCGCTCCA
CCNA2	GCAAACAGTAAACAGCCTGCG	TCAACTAACCAGTCCACGAGG
Top2A	CGCCTCCCTAACCTGATTGG	ACCGTCTCCGTCCAGAAGAA
AURA	TCTAGTCCTCCTTAACCACTTATCT	GAC ACA TGG CCT CTT CTG TAT C
p21	GGATGTCCGTCAGAACCCAT	CCCTCCAGTGGTGTCTCGGTG
PRSS2	GTTGCAGCTGCTGTTGCTGCC	TGTCATTGTCCAGAGTCCGGC
CELA3A	GGTCCCCTACAGCTGGCCCT	GCCAGAAACAAAGCTGGTCACACC
CPA2	AGTGGGTTACACAAGCTACG	AGAGGCTTCCAGATACCTTG
E47	GAGGAGAAAGACCTGAGGGAC	ACCTGACACCTTTTCCTCTTCT
Id1	AATCATGAAAGTCGCCAGTG	ATGTCGTAGAGCAGCACGTTT
Id2	ATGAAAGCCTTCAGTCCCGT	TTCCATCTTGCTCACCTTCTT
Id3	TCATCTCCAACGACAAAAGG	ACCAGGTTTAGTCTCCAGGAA
Id4	TGAACAAGCAGGGCGACA	CGTGCAAAGAAAGAATGAAAG
Mist1	CCAGCACTACCAGCAGCA	AGGACTGGGCGCTAGGTG
Dec1	GGATCTCCTACCCGAACA	TCTCCCTGACAGCTCACC
SREBP1	GCTGCTGACCGACATCGAA	GGGTGGGTCAAATAGGCCAG
CENPA	GATTCTGCGATGCTGTCTGG	CATGGTTGGTTCGCTAAACTGC
ORC1	CGATTGGCGCGAAGTTTTCT	CTTGTGGGGTAGTGTGCCAT
CDC6	CCGTAACCTGTTCTCCTCGT	TAGGTTGTCATCGCCCAGAC
DHFR	CATGGTTGGTTCGCTAAACTGC	GAGGTTGTGGTCATTCTCTGGAAATA
E-cadherin	GCCCCGCCTTATGATTCTCTG	CTCGCCGCCTCCGTACATGTC
Vimentin	GACAATGCGTCTCTGGCACGTCTT	TCCTCCGCCTCCTGCAGGTTCTT
Slug	TGTTGCAGTGAGGGCAAGAA	GACCCTGGTTGCTTCAAGGA
Twist	GAGTCCGCAGTCTTACGAGG	CTGCCCGTCTGGGAATCACT
cMYC	CCT ACC CTC TCA ACG ACA GC	CTC TGA CCT TTT GCC AGG AG
ATOH8	CAGGTGCCGTGCTACTCATA	AGTCACTCCTTGCGCTTCTT
HES1	GACAGCATCTGAGCACAGAAATG	GTCATGGCATTGATCTGGGTCAT
